# A Case of Catheter-Associated Urinary Tract Infection by Pseudomonas mendocina in the Setting of COVID-19

**DOI:** 10.7759/cureus.68900

**Published:** 2024-09-07

**Authors:** Angela Penney, Camille Ng, Nathaniel Sands, Ritu Gaikwad, Mutaal Akhter

**Affiliations:** 1 College of Medicine, California Northstate University, Elk Grove, USA; 2 Department of Internal Medicine, Mercy General Hospital, Sacramento, USA

**Keywords:** catheter-associated uti, covid-19, obstructive uropathy, pseudomonas mendocina, uti

## Abstract

Pseudomonas mendocina, a Gram-negative, aerobic bacillus, has rarely been implicated in human infections. This report details the first documented case of catheter-associated urinary tract infection (CAUTI) caused by P. mendocina in a COVID-19-positive patient. We present the case of a 70-year-old male with obstructive uropathy who presented with altered mental status eight days after testing positive for COVID-19. Urine cultures identified P. mendocina and Enterococcus faecalis. The antibiotic regimen was adjusted to cefepime and fosfomycin for coverage against P. mendocina and E. faecalis, respectively, resulting in the patient's improvement and discharge with an outpatient course of ciprofloxacin. However, the patient was readmitted 12 days later for recurrent symptoms and traumatic catheter removal, requiring nephrostomy tube placement. Follow-up revealed severe bladder and prostate abnormalities, confirming a complex interplay of factors contributing to his infection. P. mendocina is a rare opportunistic pathogen, often occurring in patients with pre-existing health conditions. This case is significant as the first CAUTI caused by P. mendocina and highlights potential links between COVID-19 and increased susceptibility to bacterial infections. The patient's comorbidities, particularly obstructive uropathy, and prolonged catheter use, were likely major factors in his infection. The role of COVID-19 in facilitating bacterial colonization remains speculative but warrants further investigation. This case report underscores the need for heightened clinical awareness and prompt intervention in patients with similar risk factors. The successful treatment regimen provides a valuable reference for managing such infections. Further research is needed to explore the interplay between viral and bacterial infections, particularly in the context of COVID-19.

## Introduction

Pseudomonas mendocina is a Gram-negative, aerobic bacillus that was first discovered in 1969, with its first documented human infection in 1992. The species originates from the Pseudomonaceae family and is commonly associated with water and soil samples [1]. Although P. mendocina shares some characteristics with its more renowned relative, Pseudomonas aeruginosa, it is a distinct pathogen with its own unique characteristics, some of which we will discuss in this report.

While infections with P. mendocina in humans are rare, the resulting infections with this bacterium are severe and have been reported to result in acute hospitalization. Documentation of previous human infections is limited. Excluding this case, there has only been one previously documented case of P. mendocina-related urinary tract infection [2]. This report presents the first case of a catheter-associated urinary tract infection (CAUTI) secondary to P. mendocina in a COVID-19-positive patient, contributing to the limited literature on P. mendocina infections in humans.

## Case presentation

**Figure 1:**
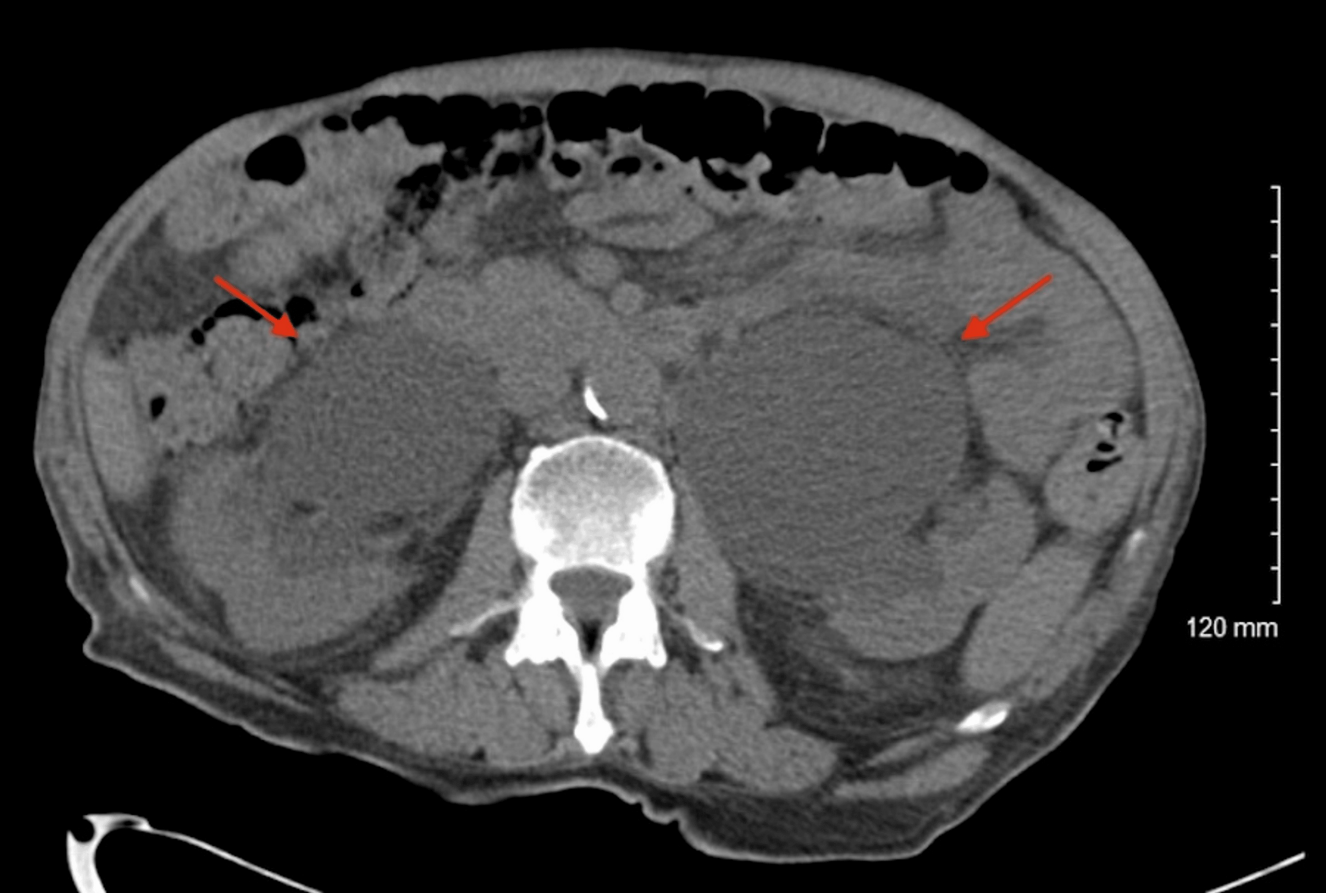
Abdominal CT image on admission revealing marked bilateral hydronephrosis and hydroureter (red arrows).

A 70-year-old male with obstructive uropathy secondary to benign prostatic hyperplasia (BPH) with an indwelling Foley catheter presented to the emergency department (ED) with altered mental status in June 2023. He had tested positive for COVID-19 eight days prior while residing at his assisted living facility. Additional past medical history includes hypertension, a recent hemorrhagic stroke in May 2023, chronic kidney disease (CKD), and encephalopathy with dementia. Upon initial evaluation in the ED, laboratory results revealed an elevated white blood cell count of 11.5 K/uL and creatinine of 4.49 mg/dL with urinalysis positive for leukocyte esterase, proteinuria, and hematuria. Empiric coverage with ceftriaxone was promptly initiated while awaiting culture results, and the Foley catheter was replaced. CT imaging revealed marked bilateral hydronephrosis and hydroureter, reportedly consistent with the patient’s chronic history of prolonged urinary obstruction (Figure 1). Additional findings included the presence of a mass in the urinary bladder lumen, later confirmed to be a segment of an enlarged prostate.

Blood cultures yielded negative results, while urine cultures demonstrated the presence of P. mendocina at a concentration exceeding 100,000 colony-forming units (CFU)/mL and E. faecalis within the range of 10,000-49,000 CFU/mL. Antibiotic susceptibility testing revealed P. mendocina exhibited sensitivity to cefepime (Table 1), and E. faecalis displayed sensitivity to ciprofloxacin and fosfomycin. Accordingly, his antibiotic regimen was modified to cefepime and fosfomycin. The prescribed course of cefepime and fosfomycin was administered during the patient’s hospital stay over the subsequent week and the patient was discharged with seven days of ciprofloxacin as an outpatient regimen, completing a total of a 14-day antibiotic course.

**Table 1:** Antibiotic susceptibility profile of this isolated strain of P. mendocina.

Antibiotic	Susceptibility/Resistance
Aztreonam	Susceptible
Cefepime	Susceptible
Gentamicin	Susceptible
Piperacillin/Tazobactam	Susceptible
Tobramycin	Susceptible
Trimethoprim/Sulfamethoxazole	Susceptible
Ceftriaxone	Susceptible

Twelve days later, he re-presented to the ED, following traumatic self-extrication of his Foley catheter, and was readmitted in the setting of anemia, altered mental status, acute kidney injury (AKI), and suspicion of UTI recurrence. Imaging studies revealed persistent severe bilateral hydroureteronephrosis without obstructing calculi, and an elevated creatinine level of 4.1 mg/dL. Seroquel was introduced into the medication regimen to mitigate the patient’s agitation and prevent continued self-extrication episodes, while empiric treatment for a possible UTI was initiated with a single dose of vancomycin and scheduled cefepime. Despite pharmacologic sedation efforts, the patient exhibited persistent catheter removal behavior, necessitating bilateral nephrostomy tube placement. Urine cultures returned negative and antibiotics were discontinued. The patient was discharged with instructions to follow up in the urology clinic for continued management of nephrostomy tubes and more definitive management of his obstructive BPH.

A cystoscopy conducted in the outpatient urology clinic revealed an enlarged nodular prostate exceeding 60 grams, alongside an overdistended bladder containing 450 cc of turbid urine. His bladder exhibited severe trabeculation with multiple cellules and diverticula. These findings supported the presence of a neurogenic bladder component (likely from his history of recent stroke) in addition to the pre-existing obstructive uropathy. Any further long-term follow-up data pertaining to this patient's clinical status is still pending.

## Discussion

P. mendocina is a rare bacterial pathogen known to cause sporadic cases of opportunistic infection, often leading to severe disease in both immunocompetent and immunocompromised patients. Of the 20 documented cases of P. mendocina infection, only a few are suspected to have been acquired outside of the hospital, and the majority arose in the setting of pre-existing health complications [1]. Similar to many previously documented cases, our patient presented with numerous underlying problems, including obstructive uropathy, chronic in-dwelling catheter, recent COVID-19 infection, and neurological factors. Notably, our patient represents the first documented case of CAUTI caused by P. mendocina, the second documented case of P. mendocina in the setting of COVID-19 infection, and just the sixth documented case of P. mendocina infection in the United States (US).

Of the six documented cases in the US, this case stands alone as the only CAUTI among one case of endocarditis, three cases of bacteremia, and one other non-catheter-associated UTI (Table 2). However, our patient shares many characteristics with preceding patients. First, every infection occurred in male patients aged 50 years or older with comorbidities that likely predisposed them to infection. Second, all of these patients were successfully treated with penicillin, fluoroquinolone, or cephalosporin antibiotics without notable problems associated with antibiotic resistance. Third, all of these infections occurred within the last eight years, a relatively recent timeframe.

**Table 2:** Previously reported P. mendocina cases in the United States.

Year	Author	Age	Sex	Comorbidities	Infection type/location	Antibiotics used	Outcome
2016	Rapsinski et al. [3]	57	Male	Gout, chronic alcohol use	Infective endocarditis	Piperacillin-tazobactam, ceftazidime (concern for cefepime related encephalopathy)	Discharged to outpatient care (44-day hospital stay)
2019	Gani et al. [4]	63	Male	Resistant HIV/AIDS	Bacteremia	Piperacillin-tazobactam (TMP-SMX for concurrent infection)	Discharged to the nursing facility with 6-week antibiotic therapy
2020	Goldberg et al. [5]	72	Male	End-stage renal disease, IgA nephropathy, AFib, heart failure with reduced EF, obesity, chronic venous stasis	Bacteremia	Ciprofloxacin	Discharge to home with home health services and follow-up
2021	Ezeokoli et al. [6]	81	Male	CAD, AFib, heart failure, CKD, DM2, CVA	Bacteremia	Cefepime, ciprofloxacin (prophylactic)	Stable discharge to subacute rehabilitation
2022	Vo et al. [2]	83	Male	DM2, hypertension, CAD, prostate cancer, COVID-19 pneumonia	Urinary tract infection	Ceftriaxone, cefepime	Complete resolution of urinary symptoms, discharge at 15 day stay
2023	This report	70	Male	BPH, COVID-19 infection, hypertension, CVA, CKD, encephalopathy with dementia	CAUTI	Cefepime, fosfomycin and ciprofloxacin (for concurrent infection)	Stable discharge to skilled nursing facility

Predisposing factors for infection

The source of our patient’s P. mendocina infection remains elusive and prompts an exploration into potential contributing factors. Our patient experienced a COVID-19 infection eight days prior to hospitalization for UTI, introducing a noteworthy element to the clinical scenario. Considering the emerging understanding of the intricate relationship between viral infections and subsequent bacterial complications, we propose that the patient’s recent encounter with COVID-19 may have played a role in predisposing him to infection with this rare microorganism. Given the understanding that irritation to tissues by viral infection can increase susceptibility to bacterial infection [7], and that SARS-CoV-2 may infect any tissues widely expressing ACE-2 receptors, including the kidneys, testes, and bladder [8,9], the postulation that our patient experienced SARS-CoV-2 infection-related tissue damage that predisposed him to P. mendocina colonization in the urinary tract is appropriate. Indeed, viral, bacterial, and fungal co-infections are common and often the cause of mortality in COVID-19 patients, although investigations into non-respiratory co-infections are less common [10-13]. Moreover, P. aeruginosa infections may occur in virtually any tissue in the body, especially when host defenses are impaired [14].

Although viral-bacterial co-infections and secondary infections are well documented, the pathophysiologic interactions between viral and bacterial agents with the immune system are incompletely understood. At the outset of the COVID-19 pandemic, suspicions about SARS-CoV-2 as a predisposing factor to opportunistic infections gained attention, but even as the pandemic has progressed, many answers have remained obscure. Importantly, opportunistic infections have appeared in situations in which immunosuppressant medications were administered to quell cytokine storm syndrome caused by COVID-19. Moreover, multidrug-resistant bacterial co-infections are suspected to arise when antibiotics are administered in the treatment for viral pneumonia, especially COVID-19. Zhu et al. reported a high rate (94.2%) of viral, bacterial, and fungal co-infection in a cohort of COVID-19 patients in China [13]. However, while the mechanisms by which viruses increase susceptibility to bacterial colonization have been ascertained, how SARS-CoV-2 infection in particular might enhance the bacterial colonization of the urinary tract is not well understood.

Interestingly, our case, and the most recently reported case of P. mendocina in the US, occurred within two years of each other, and they share some striking features, including recent COVID-19 as a predisposing factor. For example, the previous case occurred in an elderly patient being treated with corticosteroids for metastatic prostate cancer [2]. Our patient, an elderly male much like the former, had a history of obstructive uropathy secondary to benign prostatic hyperplasia. However, unlike the prior patient, who was chronically administered immunosuppressive medications, our patient, although with some degree of immunosuppression due to advanced age and recent infection, had no chronic history of significant immunosuppression. Nonetheless, this parallel observation suggests a potential association between COVID-19 and co-infections of the urinary tract.

The underlying urinary tract pathology in our patient, particularly his obstructive uropathy secondary to BPH, further elevated our patient’s susceptibility to infection. Our patient had prostate enlargement significant enough to require an in-dwelling catheter, which comes with an inherent vulnerability to infection. Historically, urinary tract infections, including those caused by Pseudomonas species, have been introduced on urinary catheters or in irrigating solutions, and underlying complications are common [14]. Periurethral and intraluminal entry into the urinary tract are also possible [15]. Our patient’s indwelling urinary catheter was noted to have been removed repeatedly both prior to and during his admission to the hospital. The association between increased indwelling time and the occurrence of UTIs has been well-established [15,16], but the association between repeated catheter replacement and UTIs has not. Combined with the possible contributions of his COVID-19 infection, our patient’s chronic indwelling catheter remains the most significant explanatory factor in the etiology of this CAUTI. Additionally, we suspect that the trauma caused by repeated indwelling catheter removals may have further contributed to the introduction of P. mendocina into our patient’s urinary tract. We call for further investigation into this relationship.

Treatment regimen

The treatment of P. mendocina and concurrent E. faecalis infection in our patient involved a combination of empiric ceftriaxone and then cefepime, fosfomycin, and outpatient ciprofloxacin, which ultimately resolved the patient’s UTI. Standard antibiotic susceptibility profiles for P. mendocina include the typical antipseudomonal agents, along with some additional agents, such as ceftriaxone and trimethoprim/sulfamethoxazole [4]. We hypothesize that, due to the novel nature of P. mendocina, there is a lower incidence of antibiotic resistance, which certainly highlights the underlying mechanism of resistance development. This also helps underscore the critical importance of employing judicious prescription practices to prevent escalation of resistance like that seen in multi-drug resistant P. aeruginosa infections.

## Conclusions

This report delves into the unique case of a rare bacterial species, P. mendocina, and its infection of the urinary tract in a patient recently infected with COVID-19. The confluence of predisposing factors to urinary tract infection, such as BPH with obstructive uropathy and an indwelling Foley catheter, creates a multifactorial interplay that we speculate likely facilitated the patient’s acquisition of this rare infection and advocates for further investigation to unravel the complexities of bacterial infections in the context of viral illnesses, particularly those associated with COVID-19. Detailing the antibiotic regimen used to successfully eliminate the bacterium, we hypothesize that the increased susceptibility of this microorganism to antibiotics may stem from its novelty. Given the rare occurrence and sparse documentation of human infection with this pathogen, this report provides valuable contributions to the understanding of P. mendocina infection and emphasizes the need for clinical vigilance in patients with similar predisposing factors to ensure prompt treatment and improve patient outcomes.
